# Evaluating the Safety and Efficacy of a Novel Glaucoma Drainage Device in High-Risk Adult Glaucoma Patients: A One-Year Pilot Study

**DOI:** 10.3390/jcm13174996

**Published:** 2024-08-23

**Authors:** Faisal Ahmed, Eduardo Normando, Syed Ahmed, Simrun Virdee, Ahmed Al-Nahrawy

**Affiliations:** 1 Department of Glaucoma, Western Eye Hospital, Imperial College Healthcare NHS Trust, London NW1 5QH, UK; e.normando@nhs.net (E.N.); syed.ahmed15@nhs.net (S.A.); simrun.virdee@nhs.net (S.V.); 2 Imperial College Ophthalmology Research Group (ICORG), Department of Surgery and Cancer, Imperial College, London NW1 5QH, UK

**Keywords:** shunt, tube, glaucoma drainage device

## Abstract

**Background:** We report on the 12-month safety and efficacy outcomes of a new non-valved glaucoma drainage device, the eyePlate-300 (Rheon Medical, Lausanne, Switzerland), in managing refractory glaucoma. **Methods:** A retrospective review was conducted on consecutive patients over 18 who underwent glaucoma drainage device (GDD) surgery with the eyePlate-300 after a single glaucoma consultation between February 2020 and April 2021, with at least 12 months of documented post-op follow-up. **Results:** A total of 16 eyes from 15 patients were included. Complete success was observed in 47% of patients and overall success in 83%. The mean IOP decreased from 31.5 mm Hg to 10.7 mm Hg (67% reduction from baseline), and the number of IOP-lowering drops was reduced from 3.1 to 0.7 at one year. The mean BCVA remained stable. No additional IOP-lowering surgeries were required, and no severe sight-threatening complications were noted. **Conclusions:** The initial one-year results suggest that the eyePlate could be a safe and effective device for reducing IOP in an ethnically diverse refractory glaucoma population. Further follow-up is necessary to determine the long-term safety and efficacy.

## 1. Introduction

Glaucoma drainage devices (GDDs) are typically employed for refractory glaucoma patients who continue to experience suboptimal intraocular pressure (IOP) despite undergoing medical, laser, and surgical treatments [1]. Traditionally, GDDs are reserved for high-risk patients in which standard trabeculectomy is expected to fail, such as those with a history of conjunctival incisional surgery (e.g., trabeculectomy, extracapsular cataract extraction, scleral buckling surgery, penetrating keratoplasty, trauma, or cicatricial disease) [2]. The 5-year follow-up results of the Tube Versus Trabeculectomy (TVT) study have supported the use of GDDs over trabeculectomy with mitomycin C (MMC) in patients with previous trabeculectomy and/or cataract extraction [3].

The Primary Tube Versus Trabeculectomy (PTVT) study compared outcomes when either trabeculectomy with MMC or tube shunts were used as the primary incisional glaucoma procedure. The 3-year outcomes showed that although trabeculectomy achieved a lower IOP with the use of fewer glaucoma medications compared with tube shunt surgery, there was no significant difference in the rate of surgical failure between the two groups [1].

The outcomes of these investigations have resulted in the growing use of tube shunts, even as a primary procedure, with increasing frequency. According to a survey conducted in the United States, their usage has risen from 7788 in 2003 to 12,021 in 2012. In 2016, GDDs were preferred over trabeculectomy in all surgical indications (including post-surgical glaucoma, uveitic glaucoma, and post-incisional surgery) except for patients who have not had any previous incisional surgery and those who have only undergone prior phacoemulsification [4]. A review of billing codes for glaucoma surgeries in France between 2005 and 2014 revealed a 440% increase in the use of aqueous drainage shunts overall [5].

As the interest in glaucoma drainage devices (GDDs) has grown, there is a need to compare different devices. The most widely used are the Baerveldt BG 101-350 (Abbott Medical Optics Inc., Santa Ana, CA, USA) and the Ahmed FP-7 (New World Medical Inc., Rancho Cucamonga, CA, USA).

Tube shunts are composed of a silicone tube connected to an endplate, which is a shared feature among them. When implanted in the anterior chamber, the tube drains aqueous humour into a potential space located between the endplate (which is fixed to the equatorial sclera) and a fibrous capsule that forms after the operation [6]. This process reduces intraocular pressure. Tube shunts have undergone various advancements since their creation by Molteno in 1969, including plate design and size variations, as well as different tube diameters. These shunts differ in the characteristics of their endplates, such as the size, shape, and material, and whether they have a venturi-based flow restriction mechanism [7]. The Ahmed FP-7 is a valved venturi-based GDD and the Baerveldt BG 101-350 is a non-valved implant that allows for unrestricted aqueous flow; the latter GDD requires a range of techniques to decrease the risk of early post-operative hypotony. These techniques include occluding the tube with a stenting suture that can be removed later or tying a ligature suture around the tube that dissolves over time or can be lasered in a clinic [8,9].

Two multicentre randomized controlled trials, the Ahmed Baerveldt Comparison (ABC) study and the Ahmed Versus Baerveldt (AVB) study, have compared these two implants. After pooling the results after 5 years from these studies, it was found that the Baerveldt implant group had a lower failure rate, a lower rate of additional glaucoma surgery, and a lower mean intraocular pressure (IOP) on fewer medications than the Ahmed implant group. However, the Baerveldt implant also carried a higher risk of hypotony [10,11].

GDDs offer certain advantages over trabeculectomy in addition to their increased effectiveness in eyes with previous trabeculectomy or cataract surgery. They are less affected by limbal scarring and are more resistant to surgical failure when undergoing additional ocular surgery [2,12].

Glaucoma drainage implants have a distinct set of complications, which include hypotony, elevated intraocular pressure after surgery, diplopia and motility problems, tube exposure due to conjunctival erosion, and corneal endothelial cell loss [13,14,15].

Recent advances in glaucoma drainage implants aim to lower the rate of complications and maintain efficacy by changing the design of the endplate, reducing the internal tube diameter, or adopting more biocompatible materials [16].

The eyePlate-300 glaucoma drainage implant (eyePlate, Rheon Medical, Lausanne, Switzerland) is a novel non-valved drainage device constructed entirely out of medical-grade silicone. This GDD is a component of the EyeWatch system (Rheon Medical, Lausanne, Switzerland), which consists of a magnetic flow-controlled system, which itself inserts into the anterior chamber and this control part is then attached to the eyePlate GDD. However, we did not use the EyeWatch system but used the novel eyePlate as a stand-alone GDD.

**Figure 1:**
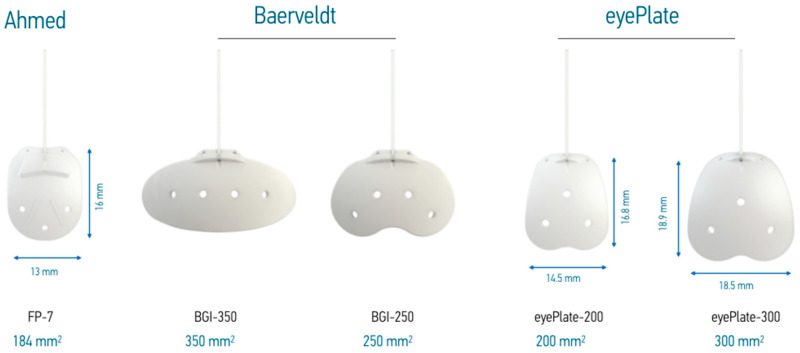
Comparison of glaucoma drainage devices with plate areas given below in blue.

The eyePlate has a convex shape with a diameter of 26 mm to contour to the curvature of the globe. It exists in two sizes, eyePlate-200 and eyePlate-300, with drainage surface areas of 200 mm^2^ and 300 mm^2^, respectively. The tube length is 30 mm with an external diameter of 0.63 mm and internal lumen diameter of 0.30 mm, which is slightly smaller than the Ahmed FP-7 and Baerveldt 250 and 350 tubes (see Figure 1). The anterior–posterior length of the eyePlate-300 is 18.9 mm, which is larger than the Ahmed FP7 at 16.0 mm, Baerveldt 250 at 13.0 mm, and Baerveldt 350 at 15.0 mm (Figure 1). The width of the eyePlate-300 is 18.5 mm which is significantly narrower than the Baerveldt 350 at 32.0 mm, but larger than the Ahmed FP7 at 13.0 mm; despite its comparatively narrow width, it still maintains a large plate area due to its relatively square shape. Its diameter also has the advantage that it does not need to be placed under the recti muscles, unlike other implants (Table 1).

**Figure 2:**
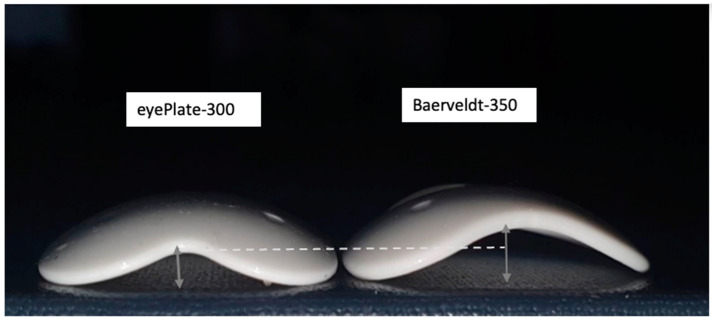
Comparison of eyePlate-300 on the left side with the Baerveldt 350 on the right. The grey double-ended arrows highlight the maximum base to height distance which is greater in the Baerveldt plate (Arrows: plate height, dotted lines show difference in plate heights between eyePlate-300 and Baerveldt-350) (image courtesy of Faisal Ahmed).

Additionally, the eyePlate-300’s endplate has a thickness of 0.8 mm, which is slightly less than the Baerveldt (250 and 350) at 0.9 mm and significantly less than the Ahmed FP7 at 2.1 mm. Its base-to-height plate profile is also lower, as depicted in Figure 2. One advantage of the eyePlate-300 endplate is its high flexibility and foldability in comparison to the Ahmed FP7 and Baerveldt 350, enabling it to lie flatter on the sclera and requiring less tension while closing the conjunctiva, and it can be implanted through smaller or radial incisions.

## 2. Materials and Methods

A non-comparative, retrospective study was conducted on 15 patients who received the eyePlate-300 glaucoma drainage implant at the Western Eye Hospital in London between March 2020 and April 2021. The Imperial College New Interventional Procedures Committee approved the study and the study adhered to the principles of the Declaration of Helsinki and its later amendments. All enrolled participants were over the age of 18 years and signed the consent form for their procedure. The participants were monitored for a period of at least twelve months. Patients were excluded if they declined to have the tube implantation surgery, if they chose other alternatives (such as a cyclodiode laser treatment), or if they chose to have other devices implanted.

Patients were offered eyePlate-300 implant surgery if they had moderate to advanced glaucoma according to Advanced Glaucoma Interventional Study Visual Field scores, with suboptimal or uncontrolled intraocular pressures despite maximal topical therapy and functional or structural signs of disease progression [17]. The decision to proceed with implant surgery was agreed upon by the supervising consultant and the patients according to clinical indication to reduce the intraocular pressure (IOP) to reach target levels. This device was chosen due to its foldability, and the technicality that allows the procedure to be performed under local anaesthesia with no need for sedation. During the study period, COVID-19 lockdowns and social distancing restrictions influenced the pre-operative protocol for patients requiring sedation or general anaesthesia. Patients needing these forms of anaesthesia had to follow a different pathway involving a mandatory 14-day self-isolation and a negative COVID-19 test result before proceeding with surgery through the “green pathway”. If these conditions were not met, the surgery had to be rescheduled and performed at an alternative hospital designated as the “red pathway”.

All 16 eyePlate-300 glaucoma drainage implant procedures were carried out exclusively at the Western Eye Hospital, London, UK. Prior to the procedure, the patients were administered either a sub-tenon or peribulbar anaesthetic using 2% lignocaine. Of the total number of patients, 44% (7/16) received no sedation, while 44% (7/16) were administered sedatives such as midazolam or fentanyl by an anaesthetist. Only 13% (2/16) of the patients underwent the procedure under general anaesthesia. The same surgical technique was employed in all procedures by the same operating surgeon (a senior consultant glaucoma surgeon).

A 7/0 silk corneal traction suture was used to retract the globe inferno-nasally to expose the superior-temporal quadrant. A limbal peritomy was performed in the superior-temporal quadrant.

**Figure 3:**
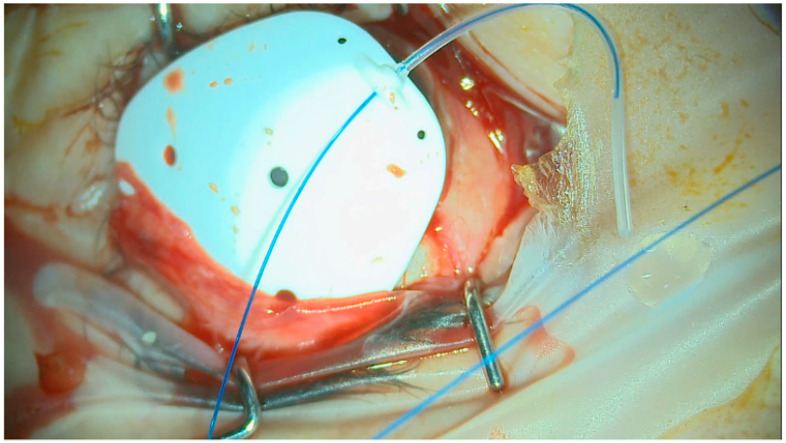
Intraoperative view of eyePlate-300 by Rheon Medical with 3/0 prolene stent inserted.

Prior to the attachment of the plate to the sclera, a 3/0 prolene suture was fed into the tube to occlude it (Figure 3). There was no need to hook the recti muscles for this technique. The eyePlate-300 was placed into the superior-temporal quadrant between the superior and lateral recti 10 mm from the limbus and sutured via the two anterior fixation holes on the plate to the sclera with 9/0 prolene.

After trimming the tube, it was placed into the anterior chamber (AC) via a sclerotomy which was placed 2 mm from limbus using a 25-gauge needle. The tube was ligated with 7/0 vicryl as an extra mechanism to reduce the flow in the early post-operative days. The tube was then sutured to the sclera with a 9/0 prolene box suture, taking care not to cause tube compression with too tight of a knot. A double layer of Pericardial Tutoplast (Innovative Ophthalmic Products, Costa Mesa, CA, USA) allograft tissue was glued with Tisseel fibrin sealant (Baxter AG, Vienna, Austria). The 3/0 prolene stent suture end was placed under the conjunctiva in the inferior fornix. The limbal peritomy was then closed with Tisseel fibrin sealant (Baxter AG, Vienna, Austria) and with 10/0 nylon sutures. No anti-scarring agents, including mitomycin C, were used for any surgeries, and topical dexamethasone was not used prior to the surgeries.

After their surgery, every patient received an orbital floor steroid consisting of 40 mg/mL methylprednisolone, along with a subconjunctival injection of antibiotic cefuroxime and dexamethasone steroid (3.3 mg/mL). Additionally, they were prescribed 0.1% dexamethasone preservative-free eye drops every two hours and 0.5% preservative-free chloramphenicol eye drops four times a day for a minimum of two weeks following the surgery.

The decision to stop glaucoma medications was tailored for each individual condition.

The baseline parameters that were recorded included age, gender, ethnicity, aetiology, previous procedures, glaucoma therapy (including the need for oral acetazolamide), past medical and ophthalmic history, visual acuity and IOP (measured by Goldman applanation tonometry), and baseline pre-operative disc and macular optical coherence tomography (OCT).

To convert visual acuity from Snellen measurements to LogMAR, we utilized the identical values used in the UK National Ophthalmology Database for Cataract Surgery. In this database, the visual acuity of Counting Fingers (CF) was assigned a value of 2.1, Hand Movement (HM) vision was given a value of 2.4, Light Perception (LP) was valued at 2.7, and No Light Perception (NLP) was valued at 3.0 [18].

The computer system for electronic patient records (Medisoft^®^, Software version 6.10, Medisoft Limited, Leeds, UK) was utilized to examine the pre- and post-operative data at intervals of 1 day, 1 week, 2 weeks, 1 month, 2 months, 3 months, 4 months, 6 months, 8 months, 10 months, and 12 months following the surgery. At each visit, visual acuity, IOP, number of topical agents, need for oral acetazolamide, steroid requirement, and central retinal thickness (HEYEX Version 2.5.5 (Build 1950), Heidelberg Engineering^®^, Heidelberg, Germany) were recorded. The patients also underwent a slit-lamp examination to assess for any abnormal clinical findings, including uveitis and conditions associated with hypotony (IOP < 5 mmHg) including shallow anterior chambers, hypotony maculopathy, or choroidal detachment. The need for further procedures was also recorded, including the removal of the 3/0 prolene stent suture or injection of Ophthalmic Viscosurgical Devices (OVDs) into the anterior chamber.

The study adhered to the World Glaucoma Association Guidelines on Design and Reporting of Glaucoma Surgical Trials to define the criterion for success [19], which was our primary outcome. Accordingly, it was considered a complete success when the intraocular pressure (IOP) was within the range of 5 mmHg to 18 mmHg or when there was a reduction in IOP of over 20% from the baseline, without the use of any IOP-lowering agents. It was considered a qualified success if IOP-lowering medications were needed to achieve the target IOP.

Failure was defined as one of the following: IOP out of target range (5–18 mmHg inclusive) or a <20% reduction in IOP from baseline on two successive post-operative visits after four weeks; the need for further glaucoma surgeries in the same eye to control the IOP; the need to use oral acetazolamide to control the IOP in the operated eye; the need to remove the implant; and severe vision loss related to the surgery (endophthalmitis, phthisis bulbi, suprachoroidal haemorrhage) or vision worsening to no light perception (NLP) due to any reason, not only glaucoma.

We also evaluated survival curves with failure defined as an IOP over 21 mmHg and less than 5 mmHg, and over 15 mmHg and less than 5 mmHg.

The study’s secondary measures comprised evaluating alterations in visual acuity, the requirement for topical medications that lower the intraocular pressure (IOP), assessing central retinal thickness (CRT) through swept-source OCT, and tracking the rate of complications.

Statistical analysis and graphs were produced using computer software, GraphPad Prism (version 9.5.1, GraphPad Software Inc., Boston, MA, USA). Descriptive statistics are described as the mean ± standard deviation for continuous variables and as a percentage for categorical variables. The Kolmogorov–Smirnov and Shapiro–Wilk tests established the normality of the results. Statistical analysis was conducted to compare the pre-operative and post-operative data using one-way ANOVA with corrections for multiple comparisons (Dunnett’s test). The results were considered statistically significant if the *p*-value was less than 0.05.

A post hoc power analysis was conducted using an estimated effect size from similar glaucoma drainage device studies. The analysis confirmed that a sample size of 15 provides a power of 80% in detecting a statistically significant difference at an alpha level of 0.05.

## 3. Results

Patients were recruited over a 12-month period from March 2020 to April 2021. There was a total of 15 patients, with 16 eyes operated on; one patient was lost to follow-up after 3 months, and hence the final population was 15 eyes of 14 patients. All patients who had received the eyePlate-300 within this period were included in the study. Only one patient had undergone cataract surgery to manage a white cataract that was obscuring vision at post-operative month (POM) 7 and endoscopic cyclophotocoagulation (ECP) was performed at the same time—primarily to reduce the number of glaucoma medications as the IOP was well controlled. One patient required YAG laser treatment to manage an iris tube incarceration at post-operative month (POM) 11.

### 3.1. Baseline Characteristics

The characteristics of our population are shown in Table 2. The population was ethnically diverse with only three (20%) patients being recorded as Caucasian. The most common diagnosis was primary open-angle glaucoma (37.5%). Two patients (12.5%) had pigmentary glaucoma, one (6.25%) had chronic angle closure glaucoma, and all the other patients had secondary glaucoma from either trauma, retinal detachment surgery, or proliferative diabetic retinopathy. All patients, except one, had undergone previous surgery to attempt to reduce their IOP; these included trabeculectomy (31.25%), micropulse diode laser trabeculoplasty (56.25%), iStent (12.5%), and cyclodiode laser (12.5%).

### 3.2. Primary Outcome Measures and Effect on IOP

For our primary outcome using the WGA IOP interval of 5 mmHg to 18 mmHg, showed two patients (13%) were classified as failures. One eye experienced vision deterioration resulting in no light perception (NLP) after undergoing pars plana vitrectomy, membrane peeling, and endo-laser treatment to manage tractional retinal detachment due to proliferative diabetic retinopathy. This occurred seven months after the eyePlate implantation. The other eye had an intraocular pressure (IOP) greater than 18 mmHg during two consecutive follow-up visits three months after surgery, although this was within target pressure.

**Figure 4:**
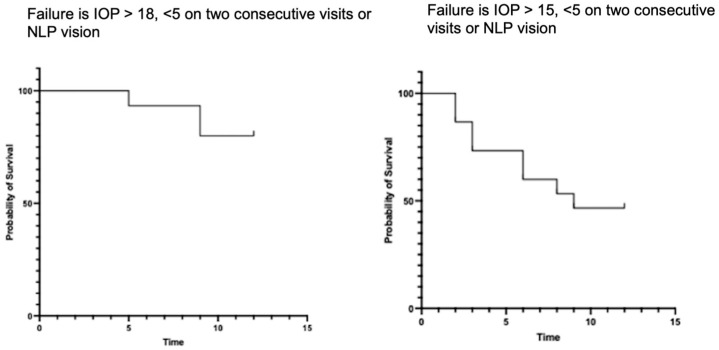
Kaplan–Meier survival analysis. Curve (**left**) shows failure, as defined by an IOP > 18 mmHg or <5 mmHg on two consecutive visits or NLP vision, and Curve (**right**) shows failure as defined by an IOP > 15 mmHg or <5 mmHg on two consecutive visits.

However, 13 eyes (87%) were considered successful as they did not require further interventions or the use of oral acetazolamide to control the IOP in the operated eye within 12 months of follow-up. Among these, seven eyes (47%) did not require any IOP-lowering drugs and were classified as completely successful. The remaining six eyes (40%) needed topical drops to lower the IOP below the treatment target and were classified as qualified success, as shown in Table 3 and Figure 4. 

**Figure 5:**
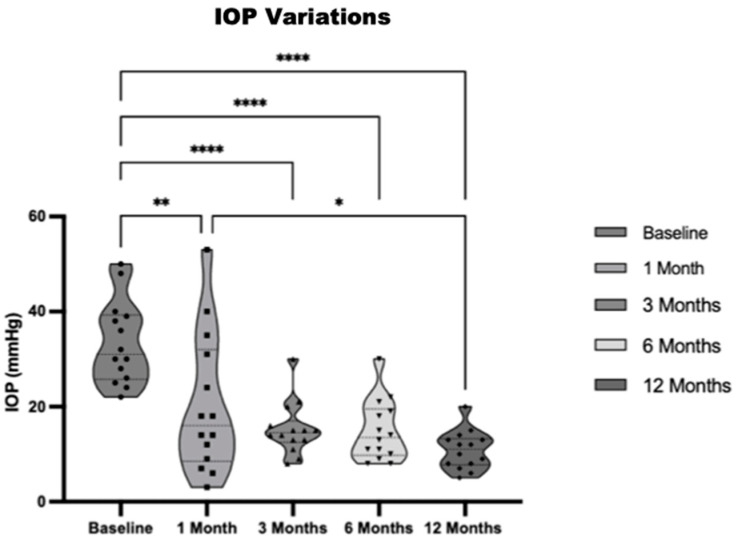
Violin plot showing mean IOP and IOP variations over the period of observation. Each symbol represents a single data point within the respective time period: **○ (circle):** Represents an individual data point at Baseline. **■ (block):** Represents an individual data point at 1 Month. **▲ (triangle):** Represents an individual data point at 3 Months. **▼ (inverted triangle):** Represents an individual data point at 6 Months. **♦ (diamond):** Represents an individual data point at 12 Months. Statistical significance is indicated by asterisks, where * *p* < 0.05; ** *p* < 0.01; ****p < 0.0001.

**Figure 6:**
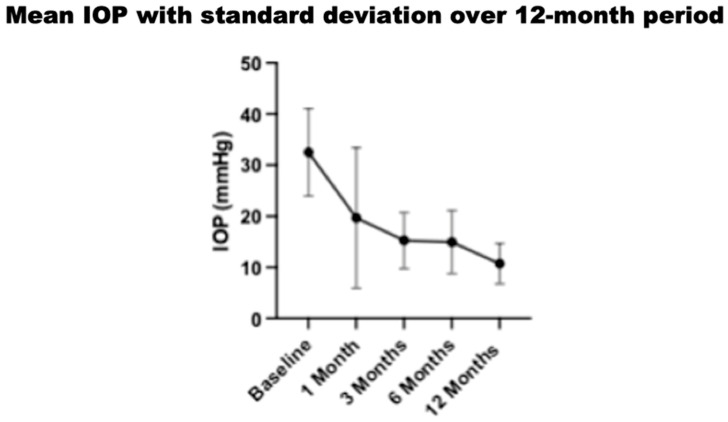
Mean IOP with standard deviation over 12-month period.

The mean initial intraocular pressure (IOP) (±SD) before treatment was 32.6 (±8.4) mmHg, with a range of 22 to 50 mmHg. Throughout the twelve-month observation period, there was a significant reduction (*p* < 0.05) in the mean IOP at all time intervals assessed. At the 1-month mark, the mean IOP decreased to 19.7 (±13.3) mmHg, reflecting a 39% reduction from baseline. At 3 months, the mean IOP further decreased to 15.25 (±5.3) mmHg, representing a 53% reduction. By 6 months, the mean IOP was 13.87 (±4.4) mmHg, reflecting a 54% reduction. Finally, at 12 months, the mean IOP was 10.7 (±3.8) mmHg, reflecting a 67% reduction from the baseline IOP. The standard deviation in IOP remained relatively low for each time period, with no increase seen at twelve months, as shown in Figure 5 and Figure 6.

### 3.3. Effect on Pressure-Lowering Drugs

Following the implantation of the eyePlate, a notable decrease in the need for IOP-lowering drops was observed at every visit. The number of topical agents showed a significant reduction (*p* < 0.05), declining from a starting point of 3.4 (±1.1) to 1.3 (±1.5) after 1 month, 1.3 (±1.4) after 3 months, 1.5 (±1.5) after 6 months, and 0.7 (±0.9) at 12 months, as displayed in Table 4 and Table 5.

A significant decrease in the necessity for acetazolamide was observed in patients, with 15 out of 16 individuals (93.8%) requiring varying doses at baseline, compared to only 2 out of 16 individuals (12.5%) requiring it after 1 month, 1 out of 16 individuals (6.25%) requiring it after 3 months, and none needing it thereafter.

### 3.4. Secondary Outcome Measures, Complications, and Retreatments

Out of the total subjects, 20% (3 individuals) underwent the removal of the stenting 3/0 prolene suture during the first four weeks, while 60% (9 individuals) had their stents removed within the first 3 months. Approximately 20% (three individuals) still had the stenting suture in place after 12 months, whereas data were not available about the tube stent of one subject (7%) who was lost to follow-up.

The baseline LogMAR visual acuity ranged from 1.14 (±0.97) to 1.26 (±0.96), which was reduced to 1.37 (±0.86) at 3 months before improving to 1.13 (±0.86) at 6 months and reaching 1.27 (±1.13) at 12 months. Out of the subjects, only one (7%) showed improvement in visual acuity at the 12-month mark compared to baseline, which was due to a reduction in the topical medication burden and an improvement in the ocular surface condition. Two eyes (13%) experienced a decline in vision at 12 months compared to baseline: one eye (7%) experienced a complete loss of vision (NLP) after undergoing vitrectomy, membranectomy, and endo-laser treatment for proliferative diabetic retinopathy (PDR), while the other patient (7%) began to develop posterior capsular opacification after undergoing phacoemulsification combined with endoscopic cyclophotocoagulation (ECP).

During the 12-month period, the central retinal thickness of the patients was evaluated using the Heidelberg Spectralis (swept source) OCT device. No statistically significant change in central retinal thickness was observed during this period. The baseline central retinal thickness was 265.60 (±65.99) µm and increased to 307.17 (±86.88) µm at one month, and then decreased to 291.10 (±51.38) µm at three months, remained at 290.67 (±63.16) µm at six months, and increased to 299.91 (±63.12) µm at 12 months, as shown in Table 6.

Within the first four weeks after the procedure, a total of five cases (31%) of hypotony were identified based on the criteria of an intraocular pressure (IOP) below 5 mmHg. One patient (7%) required an anterior chamber refill procedure with reformation using an OVD around week 2 post-operation. This was performed as an outpatient procedure using a slit lamp under 5% povidone iodine aseptic cover; 0.1–0.5 mL of 1% sodium hyaluronate (HEALON^®^) was injected intracamerally through a paracentesis incision, and covered with a one-week course of 0.5% moxifloxacin four times daily. Another patient with hypotony (7%) required a conjunctival re-suture for a supero-nasal conjunctival leak around week 2 post-operation as well; this was performed in an operating theatre, and the conjunctival leak site was sutured with 10/0 nylon sutures under topical anaesthesia. Three of these hypotony patients (20%) developed choroidal detachments, but they all fully recovered with medical treatment (1% atropine once daily) after confirming the absence of leaks, with the shortest recovery time being two weeks and the longest being three months. No cases of hypotony were observed after three months.

None of our patients required additional glaucoma surgery due to uncontrolled intraocular pressure, and no cases of diplopia were reported in the patient cohort.

Other post-operative complications included two cases (13%) of prolonged uveitis. Both patients received sub-conjunctival dexamethasone injections for resolution, and they were also treated with topical dexamethasone drops for varying durations depending on their clinical signs. Tube shortening was performed in two patients (13%) to shorten the tube in the anterior chamber as it was assessed to be close to the cornea and would pose a threat to endothelial function. One patient (7%) developed iris incarceration of the tube at 9 months post-operation, which was successfully managed with two YAG laser treatment sessions on an outpatient basis without the need for readmission to the theatre. At the end of the observation period, a note was made for each patient relating to the appearance of the capsule, the tube, and the plate. All blebs were capsulated with no tube or plate exposure at 12 months post-procedure.

## 4. Discussion

Our patient cohort predominantly comprised a very high-risk group of patients, with nearly half (40%) being Afro-Caribbean and only 20% being White Caucasian. Among them, 15 of the 16 eyes had undergone previous IOP-lowering procedures, including filtration surgery with MMC, diode, and MIGS procedures. However, despite this, our results at 12 months showed a cumulative success rate of 87% as per the definition from the World Glaucoma Association Guidelines on Design and Reporting of Glaucoma Surgical Trials using the 5 mmHg to 18 mmHg IOP interval. Unfortunately, one patient lost sight to NLP due to tractional retinal detachment secondary to diabetic retinopathy, while another patient was lost to follow-up at 3 months. The last patient had an IOP of 20 mmHg and was on one glaucoma medication; this level is below the target IOP (pre-op IOP: 48 mmHg).

Overall, none of our patients required further IOP-lowering procedures within the twelve-month follow-up period. Only one patient was reported to have been on oral acetazolamide at six months post-surgery, and this was for their other eye. The patient had their first eyePlate-300 procedure for their right eye in May 2020 and did not require any oral acetazolamide afterwards. Two weeks before undergoing the eyePlate-300 insertion in October 2020, oral acetazolamide was used for a brief period but was not required after the procedure. In May 2021, the left eye developed spontaneous hyphaema that led to a pressure spike, which required the use of oral acetazolamide for six weeks until the hyphaema resolved. His eye pressures in July 2021 were 12 and 14 for the right and left eye, respectively.

No statistically significant differences in mean pre- and post-operative central retinal thickness, as measured with OCT, were observed.

Upon analysis of the parameters used by the Ahmed vs. Baerveldt (AVB) study for defining success (IOP of 5 mmHg or above to 18 mmHg or below), our study achieved a cumulative success rate of 87% at 1 year with seven (44%) patients classified as a complete success. These results compared favourably with the results from the AVB study, where the cumulative success rate was 58% for the Ahmed GDD and 73% for the Baerveldt tube at one year [14], as shown in Table 7.

Furthermore, an analysis of the IOP interval of 5 mmHg to 15 mmHg inclusive revealed a cumulative success rate of 73%, with 40% classified as a complete success and 33% as a qualified success, suggesting that the device could potentially be used to achieve lower target pressures.

In our study, none of the patients experienced persistent complications, unlike the AVB study where 50% of the patients reported complications. Only two (13%) of our patients required early post-operative revisions, including one conjunctival re-suturing for a persistent leak and one patient for anterior chamber refill, compared to 34% of the patients in the AVB study [14].

At 12 months, none of our patients experienced complications, including persistent hypotony, uveitis, endophthalmitis, or tube complications, as described by the AVB study.

There was no statistically significant change in mean visual acuity over the 12 months of follow-up. Another patient had a permanent loss of best-corrected visual acuity (vision down to counting fingers) due to advanced glaucomatous neuropathy. And one patient had no light perception vision due to a tractional retinal detachment resulting from the progression of diabetic retinopathy.

Our results provide further evidence that non-valved GDDs can achieve lower IOPs than the standard valved Ahmed FP7 GDD. The Ahmed GDD incorporates a venturi-based, flow-restrictive mechanism designed to open between pressures of 8–10 mmHg to prevent hypotony and its associated complications. Despite these purported advantages, studies have reported a late complication of fibrous encapsulation of the plate, resulting in surgical failure. This outcome has been postulated to exist secondary to the exposure of the plate to intracameral inflammatory mediators, and result from a lack of flow restriction in the immediate post-operative period [12,13].

Comparing the 12-month outcome of this device against the results of trabeculectomy as well as Baerveldt GDD outcomes reported in the TVT study, the one-year outcome of the eyePlate-300 was similar to the trabeculectomy outcomes in terms of mean IOP and mean number of medications used, with similar overall success rates (Table 8) [3].

To explain why some drainage devices provide better drainage than other devices, several factors are believed to contribute to the lower failure rates and better reduction in IOP associated with the Baerveldt GDD. Firstly, the Baerveldt has a larger plate surface area of 350 mm^2^ compared to 184 mm^2^ for the Ahmed GDD. Studies have shown that larger plate areas are associated with lower IOPs. However, it should be noted that there have been reports of a late complication involving fibrous encapsulation of the plate, resulting in surgical failure. It has been hypothesized that this phenomenon occurs as a result of the plate being exposed to inflammatory mediators within the eye, possibly due to a lack of flow during the immediate post-operative period [12].

Previous studies assessing the biochemical properties of GDDs have demonstrated that silicone materials are associated with the least amount of inflammation post-implantation. In particular, one study [20] found silicone to be more inert than polypropylene and vivathane. The same study also found that the Ahmed glaucoma valve had a higher incidence of a hypertensive phase following surgery, which was characterized by a marked increase in intraocular pressure. Additionally, the polypropylene utilized in the Ahmed valve (Model M4) is more inflammatory than the silicone in the Baerveldt implants. In terms of clinical outcomes, the incidence of bleb encapsulation has been shown to be between 40 and 80% with the Ahmed valve and 20–30% with the Baerveldt implant [14]. Notably, higher success rates have been observed in the silicone-plate model of the Ahmed GDD [15].

In addition, it has been suggested that bleb failure in drainage devices is related to bleb height and that one possible explanation why the Baerveldt GDD may result in slightly lower pressures than the Ahmed valved GDDS is that the former GDD has a lower bleb profile due to its flatter plate shape. Indeed, increased bleb heights and volumes manifest in the hypertensive phase, the incidence of which is known to be lower in valveless implants [21,22].

Notably, the eyePlate, like the Baerveldt GDD, is made entirely of silicone and has two additional characteristics that allow it to lay flatter on the sclera. Firstly, it has a flatter plate profile than the Baerveldt GDD, and secondly, as the plate is thinner than the Baerveldt GDD and more flexible, when it is sutured to the sclera, it sits more snugly against the sclera, resulting in a very flat profile. This low plate profile may be a reason why the eyePlate tube produces favourable post-operative IOP outcomes at 12 months, even in a challenging population [12].

The Baerveldt GDD has been associated with hypotony along with choroidal effusion, suprachoroidal haemorrhage, and retinal detachments [9]. Our study reported five cases of numerical hypotony post-procedure; however, all the patients recovered, and no cases remained hypotonus beyond three months.

The results of a further analysis of complications of eyePlate-300 is shown in Table 9 and Table 10, comparing them against the results of the Ahmed and Baerveldt valves reported in the AVB and ABC trials. While differences in the complication rate may vary due to surgical experience and technique used and how far the techniques of tube implantations in general have evolved over the last decade, the relatively lower incidence of complications in the early post-operative period for the eyePlate-300 is perhaps related to the surgical experience of the operating surgeon and his technique rather due to the implant itself.

An additional advantage of the eyePlate-300 is its ability to be implanted under local anaesthesia with no need to sling the recti muscles, which can be very uncomfortable for the patient and trigger a vasovagal response in patients. This is a significant benefit because GDD implantation is commonly performed under general anaesthesia, but during the first UK COVID lockdown (2020), there was a need to limit the use of general anaesthesia due to its potential as an aerosol-generating procedure which posed a risk of COVID transmission to the anaesthetist, surgeon, and theatre staff.

Furthermore, as the procedure is performed under local anaesthesia, it reduced the need for patients to be admitted, and this was especially important considering the issues surrounding COVID, as well as the ever-decreasing number of hospital beds available. In the UK, the number of NHS hospital beds in England has more than halved over the past thirty years, from 299,000 in 1987/1988 to 141,000 in 2019/2020, while the number of patients treated has increased significantly [23].

Given the foldability of the endplate, the device could be implanted from a smaller than usual conjunctival incision, which could be helpful in eyes that had previous conjunctival surgeries.

We would also like to highlight that no mitomycin C was used for any of our tube surgeries, and the supplies of this drug have not been reliable over the last few years.

When interpreting the results of our study, certain limitations should be considered. These include a relatively short duration of follow-up and a small non randomized sample size. Additionally, visual acuity was measured with Snellen charts in the clinics and later converted to LogMAR for analysis. The study was also impacted by the restrictions imposed during the initial COVID-19 lockdown, which limited the number of pre- and post-operative tests that could be performed.

## 5. Conclusions

The management of patients with recalcitrant glaucoma is challenging. GDDs are the mainstay of surgical management for these patients, and the Ahmed and Baerveldt tubes are the most used devices. Despite being considered the gold standard, these GDDs have sub-optimal pressure-lowering effects, with many patients still requiring post-op IOP-lowering drops or other interventions after surgery, and there are associated early and late post-operative complications.

In this pilot study, we reported the first outcomes of a novel non-valved glaucoma drainage implant device, the eyePlate-300. The eyePlate-300 achieved a statistically significant reduction in IOP over 12 months (*p* < 0.05), with a significant decrease in the number of IOP-lowering medications required at 12 months compared to pre-operatively. At the 12-month follow-up, 7 of the 15 eyes (44%) did not require pressure-lowering treatment and there were only 2 failures (13%) within the first year. No further IOP-lowering procedures were required within the twelve months.

Despite the high-risk cohort of recalcitrant patients with a history of previous incisional surgery, including filtration surgery (15 of the 16 eyes had either undergone previous incisional surgery, including filtration surgery, retinal detachment surgery, or ciliary body diode laser), and who were multi-ethnic (80% non-Caucasian), the short-term success rate of the eyePlate GDD was 87%.

In addition, we did not use any antimetabolites, such as mitomycin C, in any of the surgeries, which are being increasingly used for GDD surgery and carry their own risks.

Our results suggest that the eyePlate-300 is promising with a low rate of early post-operative complications and effective IOP-lowering characteristics in the early post-operative period.

However, we emphasize the need for further long-term observations and comprehensive studies to thoroughly evaluate the safety and long-term efficacy of this innovative device.

## Figures and Tables

**Table 1:** Comparison between three different types of GDDs: eyePlate-300, Ahmed FP7, and Baerveldt 101-350.

	EyePlate-300	Ahmed FP-7	Baerveldt 101-350
**Plate size/surface area**	300 mm^2^	184 mm^2^	350 mm^2^
**Tube length**	30 mm	25.4 mm	32 mm
**Tube inner diameter**	0.30 mm	0.30 mm	0.30 mm
**Tube outer diameter**	0.63 mm	0.63 mm	0.63 mm
**Plate anteroposterior length**	18.9 mm	16.0 mm	15.00 mm
**Plate width**	15.5 mm	13.0 mm	32.0 mm
**Plate thickness**	0.8 mm	2.1 mm	0.95 mm

**Table 2:** Baseline characteristics of the study group.

**Baseline characteristic**	
Number of patients (number of eyes)	15 (16)
Mean age (SD)	59.7 years (15.8)
Gender (%)	Female: 5 (31.2)
	Male: 11 (68.8)
Ethnicity (%)	Afro-Caribbean 6 (40%)
	Caucasian 3 (20%)
	Asian 1 (6.67%)
	Other 6 (40%)
Laterality (%)	Left: 7 (43.75%)
	Right: 9 (56.25%)
Visual acuity (LogMAR)	Mean 1.14 SD 0.89 (range: 0.0 to 2.3) equivalent to Snellen 6/6—HM
IOP	32.7 (±8.4) mmHg (range: 22–50 mmHg)
**Diagnosis (%)**	
Primary open-angle glaucoma	6 eyes (38%)
Pigmentary glaucoma Chronic angle closure	2 eyes (13%) 2 eyes (13%)
Secondary Glaucomas	
Neovascular glaucoma	2 eyes (13%)
Uveitis-associated	1 eye (6%)
Post-retinal detachment surgery Undocumented	2 eyes (13%) 1 eye (6%)
**Medications Use** Mean number of topical agents used	3.43 (±1.05)
**Previous surgery or laser**	
Filtration surgery (trabeculectomy and Preserflo shunt with MMC)	5 eyes (31%)
Diode laser cyclophotocoagulation	2 eyes (13%)
Micropulse diode laser trabeculoplasty (MDLT)	9 eyes (56%)
Selective laser trabeculoplasty (SLT)	1 eye (6%)
Minimally invasive glaucoma shunt devices	2 eyes (13%)
Retinal detachment surgery/vitrectomy	4 eyes (25%)
Phacoemulsification surgery	3 eyes (19%)
Other	
Trauma repair	1 eye (6%)
Intravitreal injection (Avastin)	1 eye (6%)
AC paracentesis	2 eyes (13%)
Nil	1 eye (6%)

**Table 3:** Success rates at different IOP targets at 12 months.

	Failure	Success		
		Total	Complete	Qualified
IOP > 6–21 mmHg	0%	100%	47% (7)	53% (8)
IOP > 6–18 mmHg	13% (2)	87% (13)	47% (7)	40% (6)
IOP > 6–15 mmHg	27% (4)	73% (11)	40% (6)	33% (5)

**Table 4:** Number of subjects and topical medications used at each time point.

	Baseline	1 Month	3 Months	6 Months	12 Months
Zero Agents	1	7	8	6	7
One Agent	0	2	1	2	5
Two Agents	1	2	2	3	2
Three Agents	3	2	4	1	1
Four Agents	11	2	1	3	0
Missing Data	0	1	0	1	1

**Table 5:** Mean IOP and number of agents used at each period over the 12 months.

	IOP (SD)	Topical Agents (SD)	IOP Change from Baseline (mmHg)	% Change in IOP from Baseline
Baseline	32.7 (8.4)	3.4 (1.1)		
1 month	19.7 (13.3)	1.3 (1.5)	−12.8	−39%
3 months	15.2 (5.3)	1.3 (1.4)	−17.2	−53%
6 months	13.9 (4.4)	1.5 (1.5)	−17.6	−54%
12 months	10.7 (3.8)	0.7 (0.9)	−21.8	−67%

**Table 6:** Comparison of clinical data at pre-op, 1, 3, 6, and 12 months post eyePlate-300 insertion.

Column1	Baseline	1 Month	3 Months	6 Months	12 Months
Mean IOP mmHg (SD)	33.57 (±8.4)	19.67 (±13.3)	15.25 (±5.3)	13.87 (±4.4)	10.7 (±3.8)
Percentage of mean IOP decrease vs. baseline	NA	39.00%	53.00%	54.00%	67.00%
Mean number of topical agents (SD)	3.4 (±1.1)	1.3 (±1.5)	1.4 (±1.5)	1.5 (±1.6)	0.7 (±0.9)
Percentage of patients with IOP of 5–18 mmHg	0	63%	75%	87%	93%
Mean BCVA in LogMAR (SD)	1.12 (±1.02)	1.26 (±0.96)	1.29 (±0.89)	1.08 (±0.86)	0.96 (±0.98)
Mean central retinal thickness (um) (SD)	265.6 (±66.0)	307.2 (±86.9)	291.1 (±51.4)	290.7 (±63.2)	300 (±63.1)

**Table 7:** Comparison of results for eyePlate-300 vs. AVB outcomes (2011) [14].

	EyePlate (*n* = 15)		AVB Outcomes (2011)			
			Ahmed (*n* = 124)		BVT (*n* = 114)	
	Mean IOP	Mean Meds	IOP	Meds	IOP	Meds
Baseline	32.6 (±8.4)	3.4 (±1.1)	31.1 (±15)	3.1 (±1.0)	31.7 (±11.1)	3.1 (±1.1)
1 month	14.1 (±6.7)	0.9 (±1.3)	19.1 (±8.3)	1.1 (±1.4)	19.2 (±12.6)	1.5 (±1.6)
2 months	15.5 (±5.6)	1.3 (±1.6)	18.6 (±8.3)	1.3 (±1.4)	17.7 (±9.7)	0.9 (±1.2)
3 months	15.2 (±5.52)	1.4 (±1.5)	18.6 (±8.0)	1.5 (±1.4)	17.1 (±9.7)	0.9 (±1.2)
6 months	14.7 (±4.5)	1.4 (±1.6)	16.7 (±5.1)	1.6 (±1.3)	15.0 (±6.4)	1.0 (±1.2)
9 months	12.5 (±5.6)	1.3 (±1.5)	16.9 (±6.5)	1.6 (±1.3)	15.2 (±8.2)	1.2 (±1.3)
12 months	10.7 (±3.8)	0.7 (±0.9)	16.5 (±5.3)	1.6 (±1.3)	13.6 (±4.8)	1.2 (±1.3)

**Table 8:** Comparison between eyePlate-300 results vs. tube and trabeculectomy results from the TVT study (2009) [3].

	EyePlate-300 (*n* = 15)		TVT Study Outcomes			
			Tube (*n* = 97)		Trabeculectomy (*n* = 87)	
	IOP	Medications	IOP	Medications	IOP	Medications
Baseline	32.6 (±8.4)	3.4 (±1.1)	25.1 (±5.3)	3.2 (±1.1)	25.6 (±5.3)	3.0 (±1.2)
6 months	13.8 (±4.4)	1.5 (±1.5)	13.5 (±4.2)	1.2 (±1.2)	12.8 (±5.9)	0.6 (±1.1)
12 months	10.7 (±3.8)	0.7 (±0.9)	12.5 (±3.9)	1.3 (±1.3)	12.7 (±5.8)	0.5 (±0.9)

**Table 9:** Post-operative complications in the first year of follow-up for the eyeplate-300 vs. AVB groups [14].

Complication		One-Year Outcomes		
	EyePlate-300 (*n* = 15)	Ahmed (*n* = 124)	Baerveldt (*n* = 114)	Total (*n* = 238)
Shallow anterior chamber, *n* (%)	3 (20)	18 (15)	16 (14)	34 (14)
Choroidal effusion, *n* (%)	3 (20)	16 (13)	16 (14)	32 (13)
Iritis, *n* (%)	2 (13)	7 (6)	11 (10)	18 (8)
Persistent corneal oedema, *n* (%)	0	3 (2)	14 (12)	17 (7)
Encapsulated bleb, *n* (%)	0	14 (11)	3 (3)	17 (7)
Tube complications	1 (7)	11 (9)	13 (11)	24 (10)
Tube obstruction, *n* (%)	1 (7)	5 (4)	11 (10)	16 (7)
Tube malposition, *n* (%)	0 (0)	4 (3)	2 (2)	6 (3)
Tube erosion, *n* (%)	0 (0)	4 (3)	1 (1)	5 (2)
Cataract progression *, *n* (%)	1 (7)	5 (17)	6 (21)	11 (19)
Motility disorder, *n* (%)	0 (0)	7 (6)	3 (3)	10 (4)
Persistent hyphaema, *n* (%)	0 (0)	4 (3)	5 (4)	9 (4)
No light perception, *n* (%)	1 ^+^ (7)	1 (1)	5 (4)	6 (3)
Malignant glaucoma, *n* (%)	0 (0)	2 (2)	1 (1)	3 (1)
Suprachoroidal haemorrhage, *n* (%)	0 (0)	0	3 (3)	3 (1)
Retinal/choroidal detachment, *n* (%)	1 (7)”	0	3 (3)	3 (1)
Endopthalmitis/episcleritis, *n* (%)	0 (0)	2 (2)	0	2 (1)
Other, *n* (%)	0 (0)	15 (12)	5 (4)	20 (8)
Total No. of complications	12	107	105	212
Patients with complications, *n* (%)	6 (40%)	56 (45)	62 (54)	118 (50)

* Corrected for the number of phakic patients. ^+^ Not due to glaucoma. Tractional retinal detachment due to proliferative diabetic eye disease.

**Table 10:** Number (%) of early (≤3 months) post-operative complications in the Ahmed vs. Baerveldt comparison study [11].

Complication		ABC Study		
	EyePlate-300 (*n* = 15)	Ahmed Glaucoma Valve Group (*n* = 143)	Baerveldt Glaucoma Implant Group (*n* = 133)	Total (*n* = 276)
Tube occlusion	0 (0)	3 (2%)	12 (9%)	15 (5%)
Choroidal effusion	3 (20%)	21 (15%)	13 (10%)	34 (12%)
Suprachoroidal haemorrhage	0 (0)	0	2 (2%)	2 (1%)
Endopthalmitis	0 (0)	0	1 (1%)	1 (0.4%)
Cystoid macular oedema	3 (20%)	8 (6%)	2 (2%)	10 (4%)
Shallow anterior chamber	3 (20%)	27 (19%)	26 (20%)	53 (19%)
Hypotony maculopathy	3 (20%	5 (3%)	3 (2%)	8 (3%)
Diplopia	0 (0%)	9 (6%)	7 (5%)	16 (6%)
Corneal oedema	0 (0%)	17 (12%)	29 (22%)	46 (17%)
Tube–cornea contact	0 (0%)	7 (5%)	8 (6%)	15 (5%)
Tube erosion	0 (0%)	1 (1%)	1 (1%)	2 (1%)
Hyphaema	0 (0%)	13 (9%)	22 (17%)	35 (13%)
Vitreous haemorrhage	0 (%)	2 (1%)	3 (2%)	5 (2%)
Total No. of patients with early complications	3 (20%)	61 (43%)	77 (58%)	138 (50%)

## Data Availability

All results, tests, notes, and scans are available in the hospital’s electronic medical records and stored according to UK’s Data Protection Act (2018).
